# A model for out-of-phase boundary induced X-ray diffraction peak profile changes in Aurivillius oxide thin films

**DOI:** 10.1107/S1600576725004091

**Published:** 2025-07-02

**Authors:** Roger W. Whatmore, Debismita Dutta, Lynette Keeney

**Affiliations:** a Department of Materials, Faculty of Engineering Imperial College London SW7 2AZ United Kingdom; b School of Chemistry University College Cork Cork Ireland; c Tyndall National Institute University College Cork Lee Maltings Complex Dyke Parade CorkT12 R5CP Ireland; Oak Ridge National Laboratory, USA

**Keywords:** Aurivillius phases, perovskites, layered structures, thin films, defects, modelling, out-of-phase boundaries, X-ray diffraction, peak splitting

## Abstract

Layered crystal structures, such as the Ruddlesden–Popper and Aurivillius families of layered perovskites, have long been studied for their diverse range of functionalities. The Aurivillius family has been extensively studied for its ferroelectric properties and potential applications in various fields, including multiferroic memories. A new analytical model is presented here that explains how out-of-phase boundaries (OPBs) in epitaxial thin films of layered materials affect X-ray diffraction (XRD) peak profiles. This model predicts which diffraction peaks will split and the degree of splitting in terms of simple physical parameters that describe the nanostructure of the OPBs, specifically the structural displacement perpendicular to the layers when moving across the OPB, the angle made by the OPB at the thin-film–substrate interface, and the OPB periodicity and its statistical distribution. The model was applied to epitaxial thin films of two Aurivillius oxides, SrBi_2_(Ta,Nb)O_9_ (SBTN) and Bi_4_Ti_3_O_12_ (BiT), and its predictions were compared with experimental XRD data for these materials. The results showed good agreement between the predicted and observed peak splitting as a function of OPB periodicity for SBTN and for an XRD profile taken from a BiT thin film containing a well characterized distribution of OPBs. These results have proven the model’s validity and accuracy. The model provides a new framework for analysing and characterizing this class of defect structures in layered systems containing OPBs.

## Abstract 2

An analytical model is presented that successfully describes how out-of-phase boundaries in epitaxial thin films of layered materials affect X-ray diffraction (XRD) peak profiles. It is applied to describe the experimental XRD profiles of two types of Aurivillius oxide thin films: SrBi_2_(Ta,Nb)O_9_ and Bi_4_Ti_3_O_12_.

## 1. Introduction

Layered crystal structures have long been of interest for both academic and technological reasons. Layered silicates such as micaceous minerals have been studied for many decades and have a plethora of applications, ranging from catalysis through water purification to electronic applications such as energy harvesting. The Ruddlesden–Popper family of layered perovskite materials is of considerable academic interest, especially for use in solid oxide fuel cell cathodes. These materials exhibit a diverse range of functionalities, including superconductivity, negative thermal expansion, fast ion conductivity, hybrid improper ferroelectricity and multiferroic behaviour. Their physical properties are strongly influenced by symmetry considerations and the nature of octahedral rotation and tilt distortions (Pomiro *et al.*, 2020 ▸; Weber *et al.*, 2022 ▸; Pitcher *et al.*, 2015 ▸). In (LuFeO_3_)_9_/(LuFe_2_O_4_)_1_ superlattices, atomic-scale layering enables the coexistence and coupling of ferroelectric and ferrimagnetic order near room temperature. This emergent behaviour arises from exploitation of geometric frustration, structural distortions and epitaxial engineering (Mundy *et al.*, 2016 ▸). Similarly, in high-temperature superconducting cuprates, the layering and spacing of CuO_2_ planes play a crucial role in modulating charge-transfer energies and spatially varying electron-pair densities. The interaction between these planes and adjacent atomic layers significantly influences superconducting properties (O’Mahony *et al.*, 2022 ▸).

**Figure 1:**
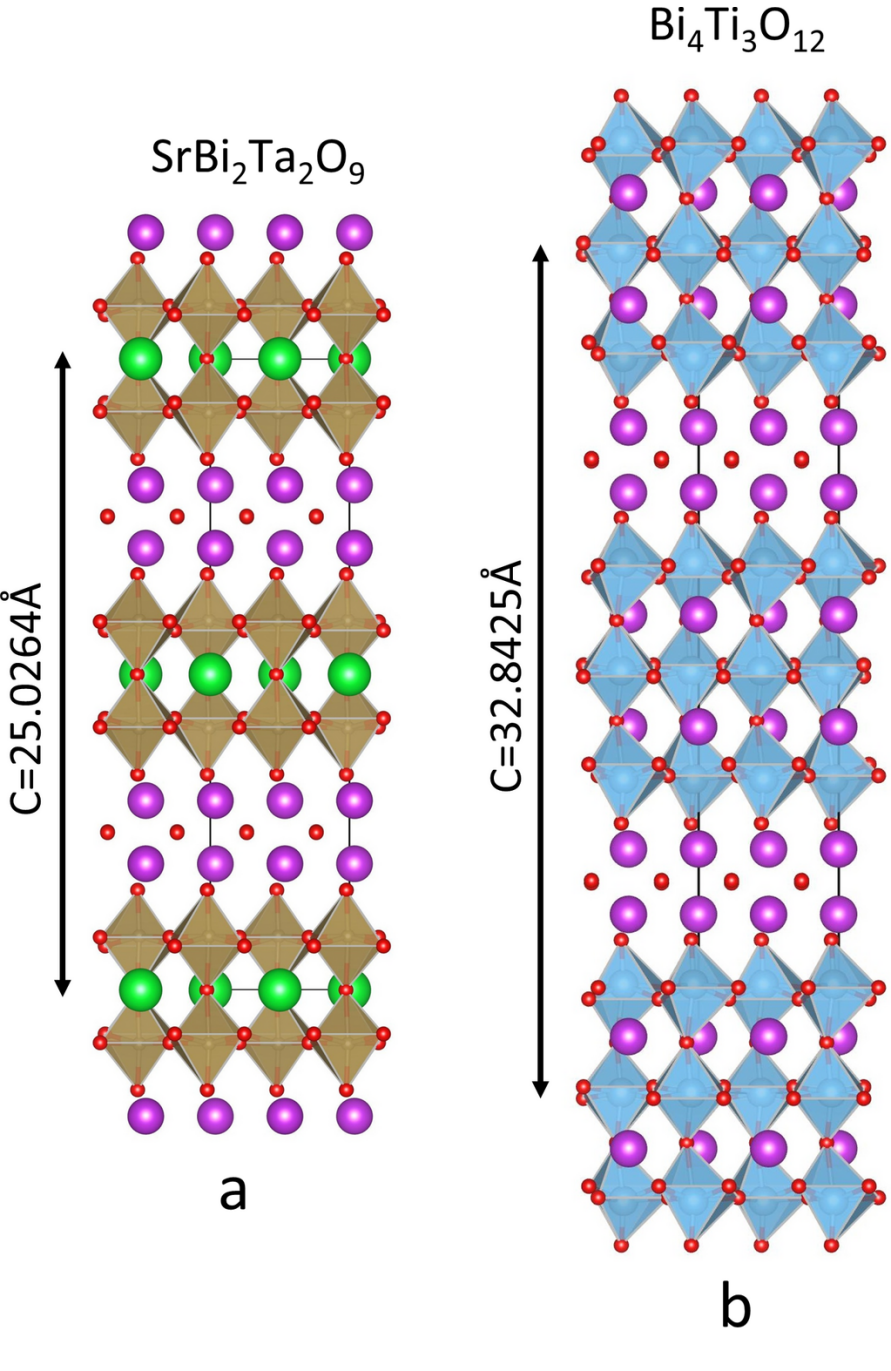
Schematic diagrams of two Aurivillius family compounds: (*a*) SBT *m* = 2 structure and (*b*) BiT *m* = 3 structure. These diagrams were created using the *VESTA* 3D visualization program (Momma & Izumi, 2011 ▸).

The Aurivillius family of layered oxide materials is very well known and has been extensively studied since its discovery (Aurivillius, 1949 ▸, 1950 ▸). The structure consists of a number of layers with the perovskite crystal structure sandwiched between [Bi_2_O_2_]^2+^ layers with the fluorite structure, and it possesses the general formula Bi_2_O_2_(*A*_*m*−1_*B*_*m*_O_3*m*+1_), where *m* denotes the number of perovskite-type layers. The structure is illustrated in Fig. 1 ▸(*a*) for *m* = 2 SrBi_2_Ta_2_O_9_ (SBT) and in Fig. 1 ▸(*b*) for *m* = 3 bismuth titanate, Bi_4_Ti_3_O_12_ (BiT).

It was realized as early as 1960/61 that BiT was ferroelectric (Van Uitert & Egerton, 1961 ▸) with a high Curie temperature of 675°C. Modifications of the compound by replacing the *A* cation with Ba, Pb or Sr, or the *B* cation with *e.g.* Nb, were quickly explored to change its properties (Subbarao, 1961 ▸). Various applications have been studied for different members of the family, including electro-optical light valves using BiT (Taylor & Miller, 1970 ▸), high-temperature piezoelectrics using Bi_3_TiNbO_9_ + 0.5 wt% CeO_2_ (Wang *et al.*, 2016 ▸) and low-fatigue ferroelectric memories using SBT (Scott & Paz de Araujo, 1989 ▸; Narayan *et al.*, 1999 ▸). The structures can easily accommodate magnetic cations such as Fe, Mn and Cr at their perovskite *B* sites, facilitating magnetic super-exchange interactions. Recently it has been shown that the Mn- and Fe-containing Bi_6_Ti_2.99_Fe_1.46_Mn_0.55_O_18_ (B6TFMO – *m* = 5) with 40% magnetic cations at the *B* sites is a rare example of a single-phase room-temperature multiferroic, displaying saturation magnetization (*M*_S_) values of 215 emu cm^−3^ and in-plane saturation polarization (*P*_s_) values of >26 µC cm^−2^ (Keeney *et al.*, 2013 ▸; Faraz *et al.*, 2017 ▸; Schmidt *et al.*, 2014 ▸). The ability to control magnetic properties with electrical polarization in such single-phase multiferroics brings with it considerable potential to apply the materials in ultra-low-energy information storage systems, potentially giving an eightfold increase in storage capacity per memory cell relative to conventional binary memory switches (Yang *et al.*, 2007 ▸; Garcia *et al.*, 2010 ▸).

**Figure 2:**
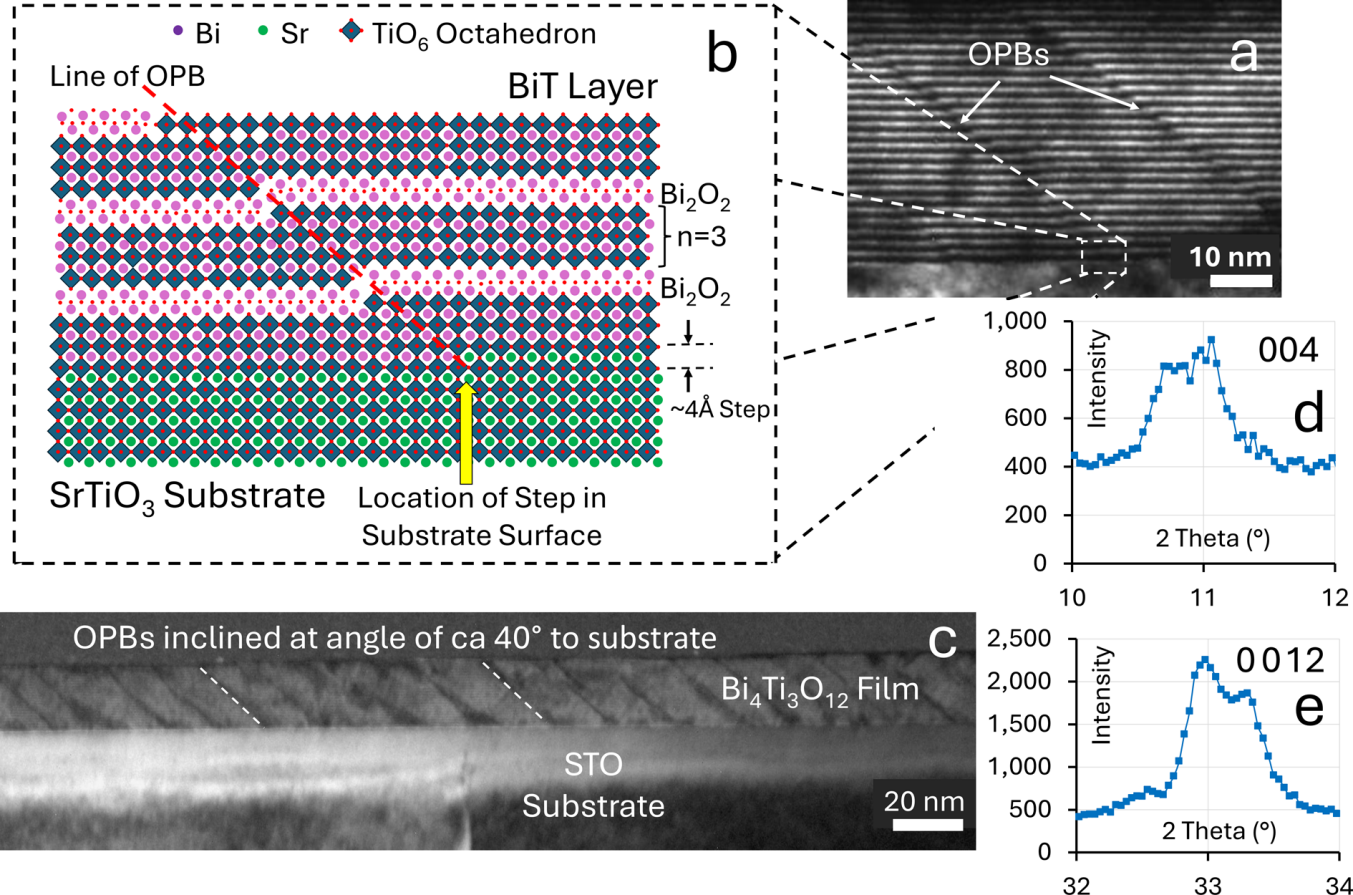
Illustrations of OPBs in BiT and their effects. (*a*) A cross-sectional TEM image of a pair of OPBs in a thin film of BiT grown by atomic vapour deposition (AVD) onto a (100) STO substrate (Deepak *et al.*, 2013 ▸). The bright horizontal lines in the image are the Bi_2_O_2_ layers in the Aurivillius structure. (*b*) A schematic diagram showing how a step at the STO–BiT interface nucleates the formation of an OPB, which displaces the atomic structure on one side of the boundary relative to the other, in this case by around 4 Å parallel to the crystallographic *c* axis. The OPB can also be associated with localized regions of higher *m*. (*c*) A higher-magnification cross-sectional TEM image of the thin film showing how the OPBs form an array with reasonably regular spacing, inclined at an angle of *ca* 40° to the substrate surface. Parts (*d*) and (*e*) show the XRD plots of intensity versus 2θ for the 004 and 0 0 12 reflections from the film illustrated in (*a*) and (*c*), showing how the 004 and 0 0 12 peaks are split [(*c*), (*d*) and (*e*) are reproduced from Deepak *et al.* (2013 ▸) with permission from AIP publishing].

The complexity of the layered crystal structures brings with it a potential for the generation of a variety of crystalline defects, including regions where the layers of the structure are out of registry. Sub-unit-cell structural defects, such as out-of-phase boundaries (OPBs) and stacking faults, commonly occur in materials with high structural anisotropy, including Aurivillius (Zurbuchen *et al.*, 2007 ▸), Ruddlesden–Popper (Kim *et al.*, 2021 ▸) and layered cuprate (Hawley *et al.*, 1991 ▸) phases. OPB crystallographic defects are illustrated in Fig. 2 ▸, in this case for BiT films grown on SrTiO_3_ (STO) substrates. They are characterized by a fractional unit-cell parameter (*c*/*x*) shift between adjacent regions along the *c* axis, manifesting as a ‘step’ within the film that is distinctly visible via transmission electron microscopy (TEM), as illustrated in Figs. 2 ▸(*a*) and 2 ▸(*c*). The atomic structure of an OPB, which is generally inclined at an angle to the substrate, is illustrated schematically in Fig. 2 ▸(*b*). The atomic structure within the region of an OPB can be complex, sometimes with localized regions of higher values of *m* occurring, although these are not a necessity. Due to the vicinal cut of the substrate, terrace structures are unavoidable on atomically flat perovskite surfaces. The resulting steps at the substrate surface can act to nucleate OPBs, making their formation a common occurrence during the epitaxial growth of layered thin films, although it should be stressed that OPBs can also occur without the specific need for substrate vicinal-step nucleation. OPBs often propagate through the entire film thickness due to their large offset and the low likelihood of opposite-sign OPB annihilation (Zurbuchen *et al.*, 2007 ▸; Campanini *et al.*, 2019 ▸). The crystallographic shear and localized regions with higher values of *m* that are characteristic of OPBs can help the structure to accommodate the compositional changes and non-stoichiometry occurring due to the presence of off-valent *B*-site dopants or the migration of volatile elements, in this case bismuth, during high-temperature growth or annealing. From a technological perspective, it has been suggested by Gradauskaite *et al.* (2022 ▸) that OPBs in thin films of the *m* = 4 Aurivillius compound Bi_5_Ti_3_Fe_4_O_15_ generated on the vicinal steps of STO substrates (Chu *et al.*, 2007 ▸) could be used in the precise control of the nucleation of ferroelectric domain walls, which could potentially be applied in the field of domain-wall nanoelectronics (Catalan *et al.*, 2012 ▸).

Stacking faults are another common type of two-dimensional planar defect caused by deviations in the regular stacking sequence of crystal planes (Kelly & Knowles, 2020 ▸). These defects can emerge during crystal growth due to substrate irregularities, form through dislocation reactions, arise from the condensation of point defects or be associated with the presence of an OPB. Crystallographic defects can have a profound impact on the properties of layered materials, influencing both their functional performance and stability. In some cases, structural defects are detrimental. For example, in the superconducting strontium ruthenate Ruddlesden–Popper phases, local defects disrupt the continuity of the layered structure, suppressing Cooper pair coherence and hindering superconductivity (Kim *et al.*, 2021 ▸). Conversely, in other materials, crystallographic defects can enhance functional properties. For instance, stacking faults and OPB defects play a fundamental role in stabilizing charged domain boundaries in the hybrid improper ferroelectric Ruddlesden–Popper phase Ca_3−*x*_Sr_*x*_Ti_2_O_7_, providing new opportunities for domain-wall-based electronic applications, including resistive switching and non-volatile memory (Nakajima *et al.*, 2021 ▸). Similarly, in the B6TFMO Aurivillius phase, OPBs and stacking faults significantly influence internal elastic strain and electrostatic energy gradients, promoting enhanced magnetic ion partitioning within the structure (Keeney *et al.*, 2017 ▸). Furthermore, these strain and electrostatic energy gradients can induce non-trivial polarization rotations near OPB defects, facilitating the emergence of charged domain walls and exotic polar vortex topologies (Moore *et al.*, 2022 ▸). Such phenomena offer exciting possibilities for next-generation energy-efficient electronics, ultra-compact data storage and ultra-high-speed data processing (Tian *et al.*, 2019 ▸).

The understanding and characterization of defects in layered structures in general and Aurivillius materials in particular is an important adjunct to understanding their behaviours and will be critical in bringing these materials to applications and for exploitation in technology. TEM allows for the identification of point defects, edge dislocations and interface structures (Nakajima *et al.*, 2021 ▸; Keeney *et al.*, 2017 ▸; Li *et al.*, 2018 ▸; Fleck *et al.*, 2022 ▸). When combined with chemical and electronic structure analysis techniques, such as energy-dispersive X-ray spectroscopy and electron energy loss spectroscopy, TEM provides insights into elemental composition and oxidation states, revealing chemical inhomogeneities linked to defects and complementing structural disorder analysis at the atomic scale (MacLaren *et al.*, 2014 ▸). However, TEM offers a limited field of view, providing highly localized information that may not fully represent macroscopic material properties. Additionally, preparing electron-transparent specimens without introducing artefacts is challenging, time consuming and costly.

X-ray diffraction (XRD) is a very important tool used for analysing the crystal- and nano-structures of materials, and a key feature of OPBs and stacking faults in Aurivillius and other layered systems is their effect on the XRD profile, where specific diffraction peaks exhibit splitting. OPB-induced peak splitting is illustrated by XRD data taken from the BiT-on-STO thin film shown in Fig. 2 ▸. In this case, there is clear peak splitting in the 004 and 0 0 12 peaks, as shown in Figs. 2 ▸(*d*) and 2 ▸(*e*).

**Figure 3:**
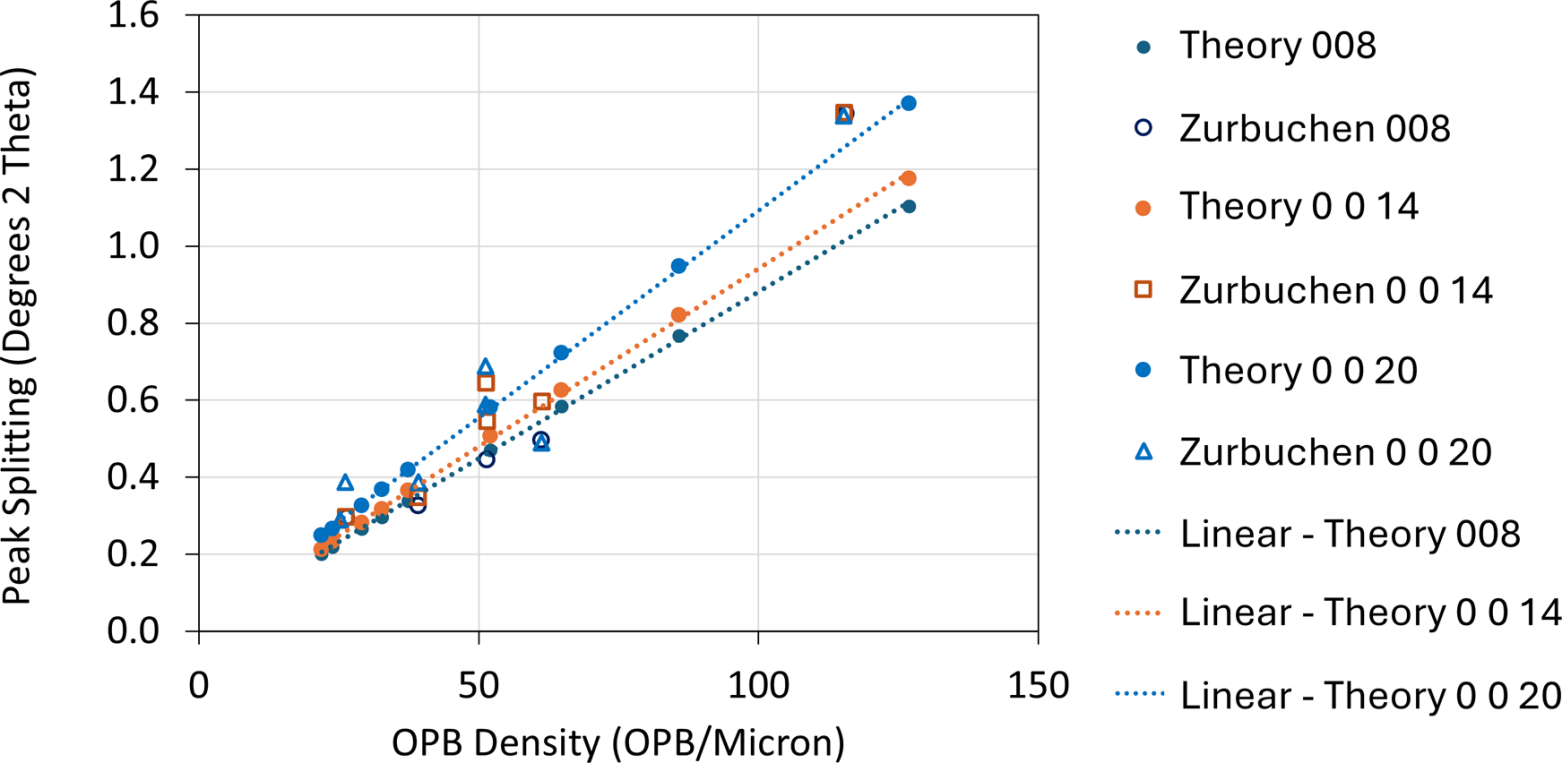
Plot of peak splitting (in degrees 2θ) versus OPB density for the 008, 0 0 14 and 0 0 20 reflections taken from thin films of SBTN. The experimental data points (open symbols) are taken from Zurbuchen *et al.* (2007 ▸). The theoretically predicted data points (solid symbols) and the corresponding straight lines are taken from the theoretical model discussed in the main text. [Experimental data points are reproduced from Zurbuchen *et al.* (2007 ▸) with permission from Springer Nature. The rights in the material from Zurbuchen *et al.* (2007 ▸) are owned by The Materials Research Society (copyright 2007), Springer Nature.]

Identifying and quantifying such peak splitting provides valuable information about the nature and density of OPBs. Zurbuchen *et al.* (2007 ▸) have reported 2θ XRD peak splitting (Δ2θ) in *m* = 2 SrBi_2_(Ta,Nb)O_9_ (SBTN) thin films and have plotted the splits in 2θ for the 008, 0 0 14 and 0 0 20 reflections as functions of the OPB density, showing a clear linear relationship between the two (see Fig. 3 ▸). Deepak *et al.* (2013 ▸) have also observed a correlation between the OPB density and the 008 XRD peak splitting in samples of BiT grown by atomic vapour deposition. Gradauskaite *et al.* (2020 ▸, 2021 ▸) observed 008 and 0 0 22 peak splitting correlated with the presence of OPBs in thin films of *m* = 4 Bi_5_Ti_3_FeO_15_.

Currently, there is no clear consensus as to which XRD peaks will be split for any given type of Aurivillius structure and defect. As Zurbuchen *et al.* (2007 ▸) pointed out: ‘*the source of the peak splitting is not entirely clear*’. In the following section, we derive a mathematical model to simulate the XRD spectrum from a film containing OPBs. This model predicts which diffraction peaks will split and provides a framework for analysing defect structures in layered systems containing this type of defect. Previous modelling efforts for defects in layered systems have primarily focused on stacking faults in macro- and micro-systems, with limited emphasis on replicating real-life defects in crystals, which are complex and do not propagate systematically within the crystal.

Matrix-based mathematical formalisms are a foundational approach to modelling defects in layered materials. Drits & Tchoubar (1990 ▸) were the first to develop a model for defects in layered silicates, to which Oueslati *et al.* (2022 ▸) and Mejri *et al.* (2022 ▸) applied a probabilistic model to describe layer-type distributions and interlamellar configurations using mixed-layer structures. This method enables structural characterization by optimizing the agreement between theoretical and experimental XRD patterns. However, it relies on statistical junction probabilities rather than direct atomistic simulations, making it less applicable to materials with localized or irregular defects. While effective for simple layered systems such as montmorillonite, it struggles with complex materials like Aurivillius phases, lithium-rich layered oxides and Ruddlesden–Popper perovskites, where disorder does not follow statistical distributions.

A refinement-based approach to XRD modelling was introduced through software with *DIFFaX* (Treacy *et al.*, 1991 ▸), specifically for stacking faults, and later with *ClayStrat* (Yuan & Bish, 2019 ▸). Both of these optimize matrix calculations to reduce computational complexity. Unlike traditional recursive methods, *ClayStrat* integrates statistical occurrence probabilities directly into diffraction intensity calculations, reducing computational complexity. This makes it more accessible for analysing complex nanocrystalline layered materials, including clays and mixed-layer structures. However, *ClayStrat* depends on predefined stacking sequences and does not inherently account for defects beyond stacking faults, such as OPBs. Additionally, while it enhances computational speed, it remains ineffective for systems where disorder is localized rather than statistically distributed. It has been extensively used for graphite, nickel hydroxide and lithium-rich layered oxides to model order–disorder transitions. More recently, the *FAULTS* program (Casas-Cabanas *et al.*, 2006 ▸), which is an extension of *DIFFaX*, was introduced to enable parameter refinement rather than simple simulation. *FAULTS* and *DIFFaX* have been particularly useful in the analysis of materials such as layered nanocomposites (Wang *et al.*, 2012 ▸; Lanson, 2011 ▸; Ramesh *et al.*, 2003 ▸), graphene layers (Dittrich & Wohlfahrt-Mehrens, 2001 ▸) and Li_2_MnO_3_ (Zheng *et al.*, 2014 ▸), where intra-layer ordering and inter-layer disordering can be discretized. However, these models remain fundamentally limited by their reliance on predefined stacking sequences. While they effectively simulate stacking disorder, they fail when applied to real-world materials where defects do not follow a single statistical distribution. For instance, in lithium-rich layered oxides, *DIFFaX* often struggles to capture the full complexity of superstructure reflections arising from Li/Mn ordering (Bréger *et al.*, 2005 ▸). Similarly, these models are ineffective for materials containing interstitial defects that do not propagate throughout the entire crystal, such as local cation vacancies or short-range phase distortions in Aurivillius phases. They also lack predictive capability for peak splitting due to OPBs.

To address the limitations of matrix-based approaches, atomistic simulations using the Debye scattering equation (DSE) have been employed for modelling powder XRD in layered nanomaterials (Leonardi & Bish, 2020 ▸; Celeste *et al.*, 2024 ▸). These models provide a more detailed description of diffraction from a set of atoms, capturing stacking disorder, in-plane misorientations and interlayer distortions. This approach has proven useful for kaolinite nanocrystals (Leonardi & Bish, 2020 ▸), where conventional Bragg analysis fails due to turbostratic disorder. However, DSE-based models require extensive computational resources, making them impractical for routine experimental use. Moreover, they rely on large-scale atomistic models, which are often unavailable for complex oxides and hybrid materials, while also lacking predictive capabilities for specific peak splitting due to OPBs. Analytical models for the X-ray scattering from defective layered materials are very useful in that they offer the prospect of more easily deriving both the crystal and defect-related nanostructures of the materials under study. A statistical model for the X-ray scattering from thin films within the Tl_1+*x*_Ba_2_Ca_2_Cu_3_O_10_ high-temperature superconducting system was derived by Holstein (1993 ▸). Kopp *et al.* (2012 ▸) derived an analytical model for the X-ray scattering from partially disordered Aurivillius sodium bismuth titanate thin films with *m* = 3 BiT containing random insertions of *m* = 4 layers of Na_0.5_Bi_4.5_Ti_4_O_15_.

The purpose of the present paper is to introduce a new analytical model to explain how OPB defects in layered materials affect the XRD peaks. This model explicitly incorporates the nanostructural characteristics of the OPBs (the step at the OPB, the OPB angle relative to the substrate plane and the average inter-OPB spacing) and accurately predicts which peaks will be split, as well as the degree of split and the relative intensities of the sub-peaks within each main Bragg maximum. This is accomplished with specific reference to the Aurivillius system, but it is believed that the model should be more generally applicable to any layer system that exhibits OPBs, such as the Ruddlesden–Popper family (Zurbuchen *et al.*, 2007 ▸).

## 2. OPB model description

### 2.1. Modelling diffraction from an array of OPBs

**Figure 4:**
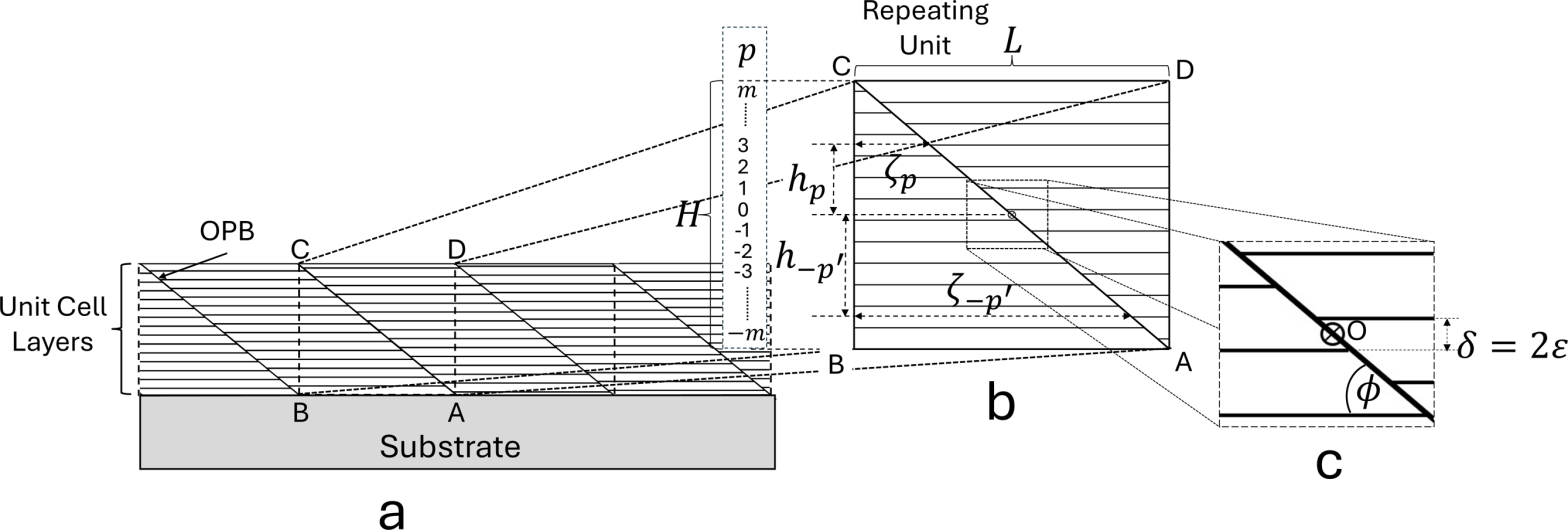
(*a*) A schematic representation of an array of OPBs in a thin film of a layer-structured material grown onto a substrate. The thin film is divided up into a set of identical repeating units. (*b*) A magnified representation of one of the repeating units indicating the dimensions used in the modelling described in the main text. (*c*) Defining the angle 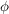 made by the OPB with the line AB (parallel to the layers of the structure) and showing the centre (O) of the region ABCD

Consider a layered material grown onto a substrate containing a regular array of OPBs that propagate through the entire film thickness, as illustrated in the schematic diagram in Fig. 4 ▸(*a*). This structure can be broken down into identical units, as outlined by the dashed lines. This unit, represented by the rectangle ABCD shown in the magnified view in Fig. 4 ▸(*b*), is repeated regularly throughout the structure and will be used in modelling its diffraction. The region ABCD contains an OPB and consists of *N* layers where 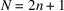 (note that this also defines the variable *n*).

The term *p* in Fig. 4 ▸ is a dummy variable that is used to count the layers up (*p* positive) or down (*p* negative). The block ABCD consists of two parts, an undisplaced part within the region defined by ABC on the left of the OPB and a displaced part on the right within the region defined by ACD. The various parameters are defined as follows.

Firstly, *c* is the *c*-axis lattice parameter and 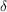 is the dis­placement of the layers at the OPB in a direction parallel to the *c* axis. We set 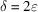.

ABCD has a height 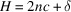 and a width *L*. The term *L* is determined by the OPB density 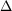, where 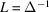. We assume that the size of the block is smaller than the coherence length (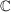) of the X-rays being used, in which case the scattered waves from the component parts of block ABCD will interfere in the far field. We can estimate 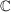 for radiation of wavelength 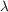 by using the equation 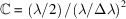 [taken from Lee *et al.* (2011 ▸)], where 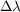 is the linewidth of the radiation source being used. The value of 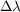 for Cu *K*α_1_ radiation (wavelength = 1.54051 Å) is 0.000461 Å. This gives 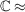 500 nm, which is much larger than the typical dimensions being considered here. Fig. 4 ▸(*c*) defines the angle 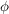 made by the OPB with the line AB (parallel to the layers of the structure) and shows the centre (O) of the region ABCD. The region has 180° rotational symmetry about O. The angle 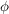 is related to the parameters *L* and *H* through the equation
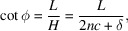
where 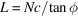 and 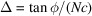.

We describe the Aurivillius layers within the undisplaced region ABC by giving them numbers (*p*) defined with reference to the centre (O) of ABCD. The layer containing O is defined as 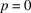 in both regions, ABC and ACD. The *p* term is increased going upwards in region ABC and decreased going downwards. The height of the centre of each Aurivillius layer relative to O is then defined as 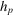. The length of each layer at the layer centre line between the line BC and the OPB is then defined as 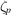. Finally, the 180° rotational symmetry of ABCD means that layer *p* in the undisplaced region ABC is identical to layer 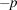 in the displaced region. We will primarily concern ourselves with the scattering for the reciprocal lattice vector perpendicular to the basal planes of the layers. This vector contains the 00*l* reflections.

The scattering from the block ABCD can be computed by adding together the scattering from each layer as we move up the block from the line AB, taking into account (1) an appropriate phase shift for each layer and (2) the fact that the length of each layer changes as we move up through the block. The scattering factor 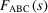 for the undisplaced layers for the scattering vector **s**, where the magnitude of the vector is 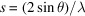 (and 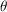 is the Bragg diffraction angle), is given by
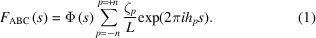

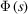
 is the structure factor for one unit cell, given by
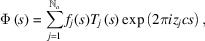
where 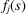 is the form factor and 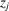 is the *z* coordinate for the *j*th atom in the unit cell. The summation is taken over all the 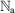 atoms in the unit cell. 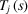 is the Debye–Waller factor or atomic displacement parameter for the *j*th atom.

Recognizing the rotational symmetry within the block ABCD, the scattering factor 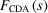 for the displaced layers for the scattering vector **s** is given by
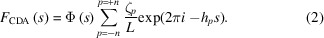
Given that ABCD is much smaller than the coherence length of the radiation, 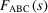 and 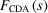 can be summed to get the total scattering factor for the block ABCD, which we write as 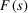:
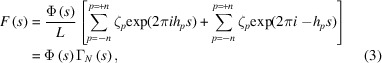
where
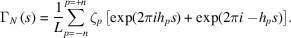
Recognizing that 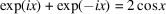, we can write
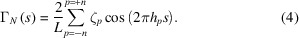
In Section S1 of the supporting information it is shown that equation (4) can be expressed as
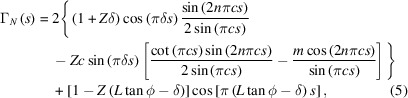
for *s * > 0, where 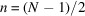 and 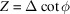.

Equation (5) only depends on the parameters characterizing the OPBs, namely the inter-OPB length (*L*), which determines the number of layers in the calculation (*i.e.* the value of *N*); the angle made by the OPB at the substrate surface (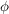); and the displacement at the OPB (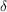).

The diffraction intensity modulation in equation (5) arises from the interplay between cosine and sine terms, which govern constructive and destructive interference, respectively. The cosine term 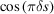 reinforces Bragg peaks when OPBs minimally disrupt periodicity, while the sine term 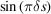 introduces oscillations that cause peak splitting by redistributing intensity into sidebands. Peak splitting occurs when destructive interference suppresses intensity at the original Bragg position, forcing the emergence of two side peaks. This behaviour directly reflects the real-space structural displacement at OPBs, where periodic phase shifts create structural discontinuities that manifest as split peaks in reciprocal space.

Once we have 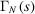 from equation (5), we can substitute it into equation (3) and calculate the intensity as a function of *s* or 2θ using the expression
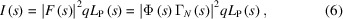
where *q* = multiplicity [= 2 for the (00*l*) reflections] and 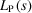 = the Lorentz–polarization factor.

This predicts the intensity 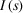 continuously as a function of *s* (or 2θ) rather than solely at the 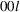 diffraction peaks.

**Figure 5:**
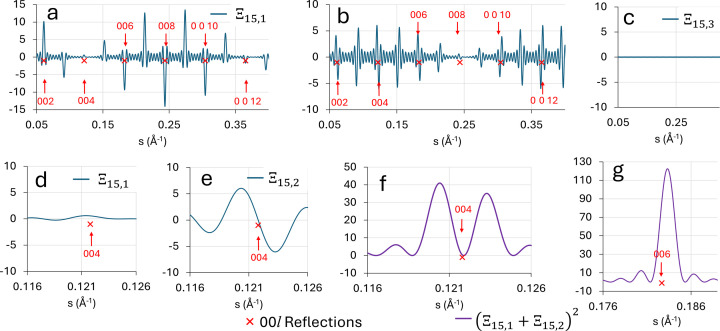
Plots of the functions (*a*) 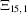, (*b*) 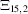 and (*c*) 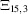, as defined in the main text, plotted for the BiT crystal structure with OPB defects: *c* = 32.8425 Å, 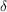 = 4 Å and *N* = 15. The positions of the 00*l* reflections where 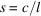 are marked with red crosses and labelled. Plots (*d*) and (*e*) show the functions 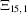 and 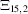, respectively, in greater detail around the BiT 004 reciprocal lattice point. Plots (*f*) and (*g*) show the function 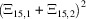 plotted around the BiT 004 and 006 reciprocal lattice points, respectively.

Equation (5) can be broken down into three components, as follows:
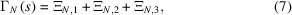
where
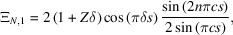

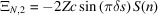
and
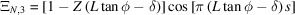
with
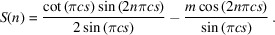
Equation (7) can be used to understand why particular diffraction peaks will be split. As an example, Figs. 5 ▸(*a*), 5 ▸(*b*) and 5 ▸(*c*) plot the functions 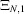, 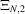 and 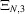 versus *s*, respectively, which make up the function 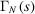 for *N* = 15 (*n* = 7) in a layered Aurivillius material possessing OPBs [in this case BiT, for which *c* = 32.8425 Å, 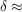 4 Å and 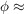 40° (Deepak *et al.*, 2013 ▸)]. It should be remembered that [from equation (6)] the diffracted intensity is proportional to 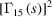. The positions of the 00*l* reciprocal lattice points where 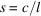 and where we would normally see a diffraction maximum (or ‘reflection’) are marked on the figures and appropriately labelled. 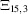 is small in comparison with 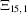 and 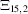 and thus can be neglected. For the regions of *s* around certain reciprocal lattice points, *e.g.* 004 and 008, where 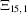 is a maximum [see Fig. 5 ▸(*a*)], 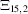 is a minimum [see Fig. 5 ▸(*b*)] and *vice versa*. This corresponds to the fact that the envelope of 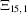 is modulated by 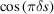, while the envelope of 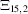 is modulated by 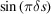. For 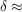 4 Å, these modulation functions have a periodicity of about 0.5 Å^−1^. This envelope modulation is clearly visible in Figs. 5 ▸(*a*) and 5 ▸(*b*). Figs. 5 ▸(*d*) and 5 ▸(*e*) plot the functions 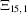 and 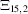, respectively, in the region of the 004 reciprocal lattice point. In this region, where *s*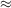 0.12, 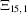 is small [this is because 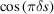 = 0 for *s* = 0.125]. On the other hand, 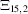, which is modulated by 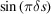 (= 1 for *s* = 0.125), goes from a strongly positive value to a strongly negative value either side of the position of the reflection. Hence, in the region of the 004 reflection, the diffracted intensity, which is proportional to 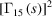, is dominated by 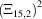 because 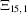 is relatively small in this region of *s*. The function 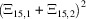 is plotted in Fig. 5 ▸(*f*) and the doubled nature of the peak is clearly visible. On the other hand, in regions of reciprocal space where neither 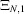 nor 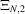 dominate we tend to see single peaks. An example is the region around 006, for which *s*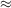 0.184. Here, the absolute values of 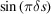 and 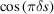 are similar in magnitude and the plot of 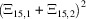 gives a single peak, as shown in Fig. 5 ▸(*g*). The next region of *s* where 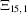 is minimized due to the 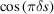 envelope modulation occurs near the 0 0 12 reflection, where 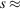 0.37 [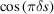 = 0 for *s* = 0.375]. Here, 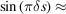 1 and the intensity is again dominated by 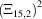, and a doubled peak is shown.

The reasons for the single or doubled peaks can be understood through a more detailed consideration of the shapes of the functions responsible for the more rapid oscillations in 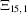 and 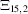 [as opposed to the more slowly varying ‘envelope’ functions 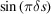 and 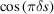]. The more rapid oscillations are driven by 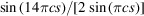 in 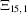 and 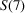 in 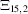, as defined in equation (7). In the region of the reciprocal lattice points where 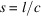, the first of these two functions equals 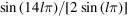 and this will give a single positive (or negative) value. On the other hand, the second function transitions from positive to negative (or negative to positive) values within the same region of reciprocal space. This means that if the first function {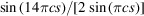} dominates 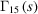, a single diffraction maximum will be seen, while if the second function [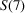] dominates 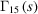, a doubled peak is produced.

Hence, doubled peaks will be seen in regions of *s* where 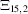 dominates the 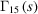 function. This will be those regions where 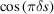 is small or close to zero, and when 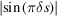 is at a maximum, *i.e.* when 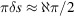, where 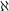 is an odd integer for 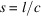, or when 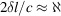.

Thus, a doubled peak is to be expected for those regions of reciprocal space where
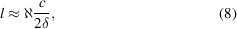
where 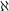 is an odd integer. It is not necessary for the predicted value of *l* from equation (8) to be an integer for that reflection to be doubled; it just needs to be around an integer value so that 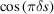 is significantly smaller than 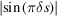. The combination of doubled reflections can also be used as a useful guide to estimate 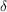 in advance of a full calculation using the model.

Equation (8) can be tested for BiT and SBT, for which reliable values of *c* and 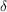 are available, as follows: for BiT, *c* = 32.8425 Å and 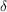 = 4 Å (Deepak *et al.*, 2013 ▸), so 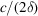 = 4.1; for SBT, *c* = 25.0264 Å and 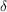 = 4.5 Å (Zurbuchen *et al.*, 2007 ▸), so 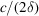 = 2.78.

Table 1 ▸ lists the values of *l* as calculated from equation (8) for which a doubled peak is to be expected, and the values of *l* for which a doubled peak is actually observed. There is good agreement between the predictions and the observations.

### 2.2. Accounting for a distribution of inter-OPB distances

It can be seen from the cross-sectional TEM image presented in Fig. 2 ▸(*c*) that the OPBs do not form a perfectly regular array as presented schematically in Fig. 4 ▸(*a*). A distribution of inter-OPB distances around some average value can be accounted for by modelling *L* with a statistical distribution of values. *L* can be modelled using with a normal distribution (although other statistical distributions may be more appropriate, as will be seen below). For a normal distribution, the probability 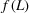 of any given value of *L* occurring is
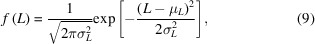
where 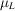 is the mean value of *L* and 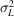 is the variance of *L*.

If there are a range of values of *L* spanning, say, ±3 standard deviations from the mean from 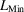 to 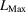, then the corresponding minimum and maximum values of *N* are 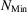 and 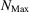. For these values of *N* and every value in between them, a value of 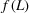 can be calculated and used to weight the corresponding value of 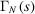 in equation (5).

These weighted values can then be summed from 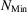 to 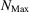 to get a new value of 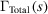 for the system:
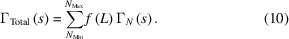
This value of 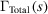 can be used with equation (6) to model the intensity diffracted from the system.

**Figure 6:**
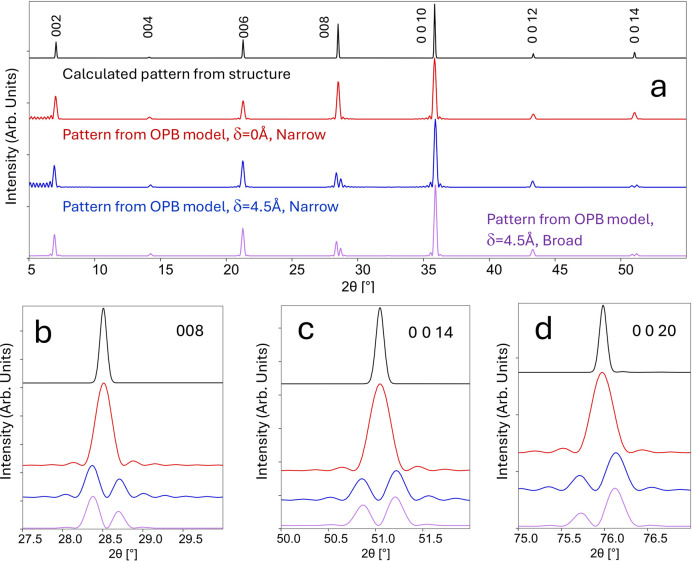
Calculated XRD patterns for SBT (using monochromatic Cu *K*α radiation). (*a*) Range from 5 to 55° 2θ. (*b*) Region around 008. (*c*) Region around 0 0 14. (*d*) Region around 0 0 20. The black (top) lines in each case correspond to a computation using the structural data published by Shimakawa *et al.* (1999 ▸) for a thin film with 90% *c*-axis preferred orientation. The red (middle) lines correspond to the computed pattern with the OPB model described in the main text, assuming an OPB angle (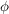) of 53° and a displacement (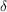) of 0 Å. The blue lines are as for the red but with a displacement (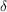) of 4.5 Å and with a narrow normal distribution for *f*(*L*), such that 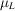 = 26.74 nm (37.4 OPBs µm^−1^) and 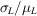 = 0.05. The purple lines are as for the blue but with a broader distribution for 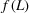, such that 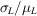 = 0.2.

It is useful now to compare the intensity calculated from this model with more conventional calculations of diffracted intensity. Fig. 6 ▸ presents the intensity diffracted as a function of 2θ from SBT using the structural data published by Shimakawa *et al.* (1999 ▸).

The first line (black) presents the pattern as calculated using the *CrystalDiffract7* software (https://crystalmaker.com/) assuming a 90%-orientated thin film. The OPB model described above was used to calculate the diffracted intensity versus 2θ for a set of different models using the OPB data for SBTN reported by Zurbuchen *et al.* (2007 ▸), setting the OPB angle (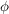) to 53°. A normal distribution for *f*(*L*) was used with 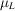 = 26.74 nm (corresponding to 37.4 OPBs µm^−1^ and *N* = 15). Fig. 6 ▸(*a*) presents the intensity diffracted from 5 to 55°, while Figs. 6 ▸(*b*), 6 ▸(*c*) and 6 ▸(*d*) concentrate on the peaks where we would expect to see splitting. Firstly, if the displacement at the OPBs is set to zero (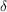 = 0 Å), then the diffraction pattern (red line) from a perfectly periodic crystal structure with no defects would be expected, and that is indeed the case – it is closely similar to the standard calculated diffraction pattern from the highly oriented thin film. The relative intensities of the reflections are the same as the conventional calculations and no diffraction line splitting is observed. This is a useful ‘confidence check’ on the model. If the OPB displacement (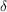) is set to 4.5 Å, and a narrow normal distribution for 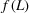 is assumed, using the same values of 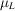 = 26.74 nm and 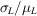 = 0.05, then the diffraction pattern indicated by the blue lines is produced. The 008, 0 0 14 and 0 0 20 lines, and only those lines, are split (as predicted above in Table 1 ▸), with some indication of weak satellites around all the lines. If a broader distribution is set for 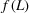, such that 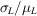 = 0.2, but keeping everything else constant, then the diffraction pattern indicated by the purple lines is obtained. In this case, the line splits are similar to those obtained with the narrower distribution, but the satellite intensities are much reduced.

## 3. Comparison of OPB model predictions with experimental observations

### 3.1. SBT

The model presented here can be used to calculate the line splitting in 2θ versus OPB density and compare the results with the experimental data presented by Zurbuchen *et al.* (2007 ▸). The results of these calculations [assuming a narrow normal distribution for 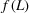 with 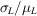 = 0.05] are presented in Fig. 3 ▸ as solid points, while the experimental points are plotted as open symbols. There is significant scatter in the experimental data, but the points calculated from the new model are in good agreement with the reported data. The sets of peak-split data for each reflection lie on their own straight line. The values of 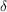 = 4.5 Å and 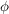 = 53° are, as reported by Zurbuchen *et al.* (2007 ▸), determined from TEM cross-sectional images of the thin films. No further adjustment of these parameters has been made to make the new model fit the data.

**Figure 7:**
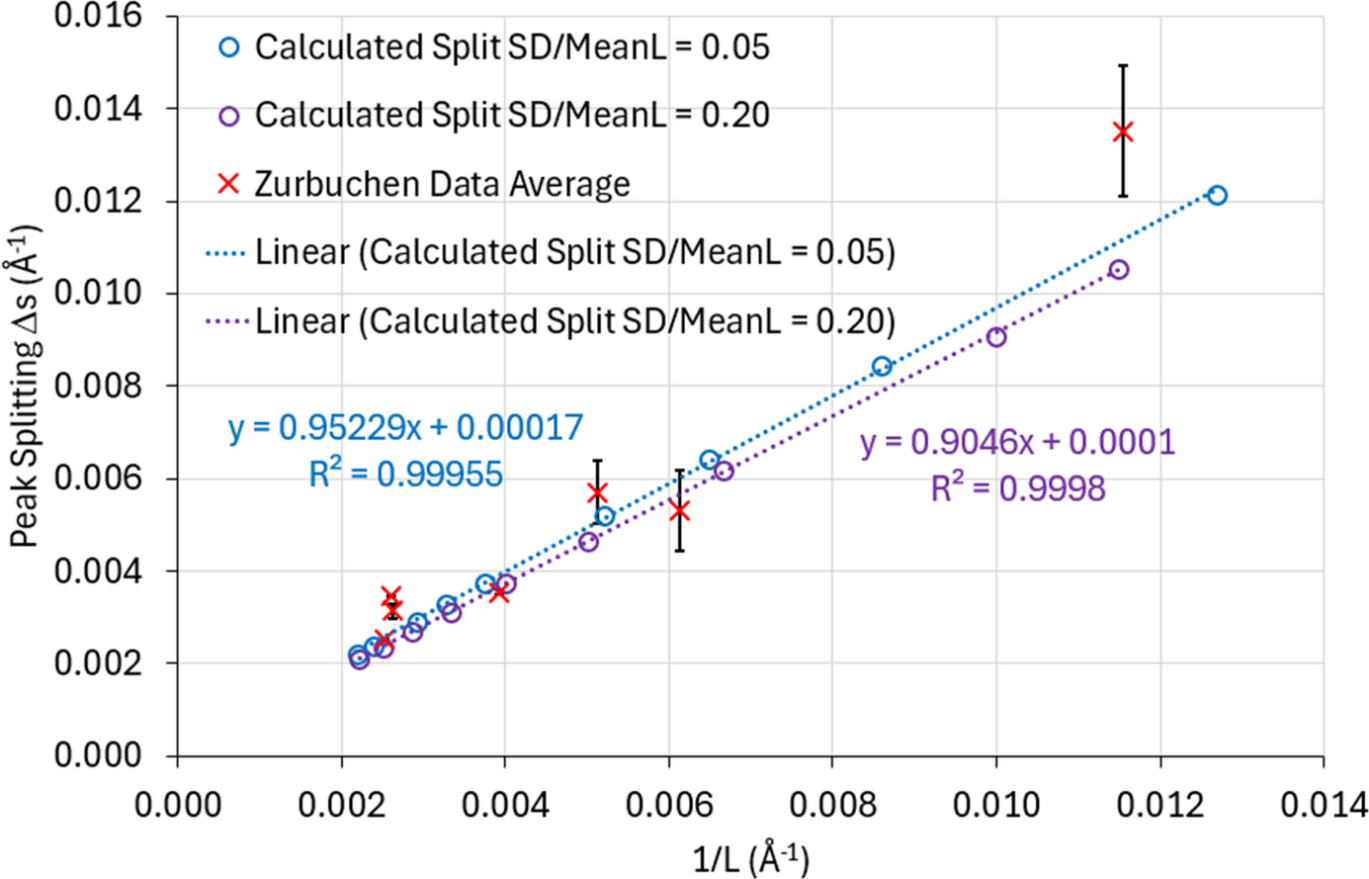
A plot of peak splitting 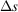 versus 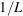 (both in units of Å^−1^). The peak splittings are taken as the average for the 008, 0 0 14 and 0 0 20 reflections from thin films of SBTN. The experimental data points (red crosses) are calculated from data published by Zurbuchen *et al.* (2007 ▸). The theoretically predicted data points (open symbols) and the corresponding straight lines are calculated using the OPB theoretical model discussed in the main text, with the structural data published by Shimakawa *et al.* (1999 ▸) and OPB parameters 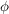 = 53° and 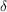 = 4.5 Å, as published by Zurbuchen *et al.* (2007 ▸). The blue points correspond to a narrow normal distribution for 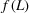, such that 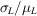 = 0.05, while the purple points correspond to a broader distribution for 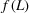, such that 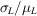 = 0.2. [Experimental data points are reproduced from Zurbuchen *et al.* (2007 ▸) with permission from Springer Nature. The rights in the material from Zurbuchen *et al.* (2007 ▸) are owned by The Materials Research Society (copyright 2007), Springer Nature.]

Equation (5) describing 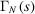 does not depend on which reflection is being observed. However, if the peak splits are measured in ‘degrees 2θ’, then each reflection gives a different line of Δ2θ versus OPB density, as in Fig. 3 ▸. On the other hand, if the peak splits are described in ‘*s*’ space (*i.e.* in units of Å^−1^), then each reflection should give a similar value of 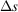. The experimental Δ2θ data from Zurbuchen *et al.* (2007 ▸) were converted into 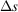 data and the peak-splitting values for a given OPB density averaged. The resulting 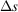 data are plotted versus 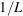 as the red crosses in Fig. 7 ▸. (The error bars on these data represent the spreads in the 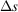 values at each value of 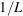.) The calculated values of 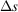 versus 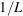 from the new model are also presented on this graph for 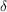 = 4.5 Å and 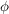 = 53°, for 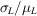 = 0.05 (blue points and line) and 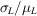 = 0.20 (purple points and line). The calculated peak splits across the three different reflections used (008, 0 0 14 and 0 0 20) differed by no more than 0.5%. The model closely describes the experimental data, with the main deviation for large values of 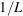 (*i.e.* a high OPB density). The statistics associated with the straight lines through the calculated data points are also presented in Fig. 7 ▸. The quality of the fit is excellent.

The line intercepts are close to the origin, as would be expected – the presence of no OPBs should give no line splitting. The line gradients are weakly dependent on the width of the 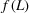 distribution used in the model.

### 3.2. BiT

**Figure 8:**
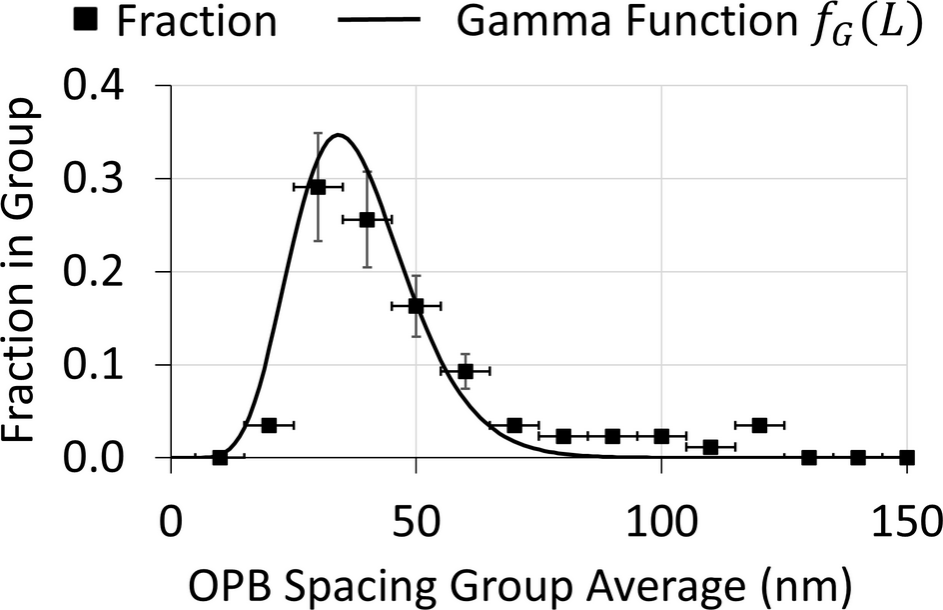
The observed distribution of 85 inter-OPB lengths measured from TEM images of a thin-film sample of BiT, plotted as fractions of lengths observed in a group 10 nm wide, centred at 10 nm intervals, compared with a gamma distribution function (see main text) using 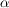 = 10, 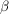 = 0.26 and *K* = 10. The mean value of *L* was set to 38 nm, which gives a mode of 34.2 nm and a standard deviation of 12.0 nm. The error bars indicate the widths of the groups (±5 nm) and the estimated error in the number fraction in each of the groups (±20%).

The thin-film sample of BiT grown on STO for which the cross-sectional TEM images are presented in Fig. 2 ▸ was examined in great detail. The BiT thin films were grown by direct-liquid-injection chemical vapour deposition using the AIXTRON AIX 200 4/FE AVD system. XRD measurements were carried out at room temperature using a Philips PANalytical MRD XRD system with Cu *K*α radiation. TEM characterization was performed using a JEOL 2100 transmission electron microscope. Full details of film growth and characterization are described by Deepak *et al.* (2013 ▸). Several cross-sectional TEM images were available and three further images are presented in Section S2 of the supporting information as Fig. S1. From these and similar images, it was possible to reliably measure 86 individual inter-OPB lengths (*L*). These were grouped into sets 10 nm wide and centred at 10 nm intervals. These data were used to plot the distribution graph shown in Fig. 8 ▸. In this case, the distribution of inter-OPB lengths (*L*) can best be described using the gamma distribution:
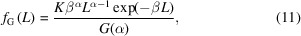
where 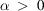 is the shape parameter, 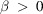 is the rate parameter, 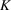 is a constant and 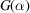 is the gamma function, defined by 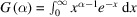.

The gamma distribution function, which has a rapid rise at the low-*L* end of the distribution and a longer tail at the high-*L* end, models the observed distribution well, and better than would be expected for a normal distribution, which is symmetrical about the mean. The parameters used in the distribution were 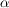 = 10, 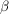 = 0.26 and *K* = 10. The mean value of *L* was set to 38 nm, which gives a mode of 34.2 nm and a standard deviation of 12.02 nm.

**Figure 9:**
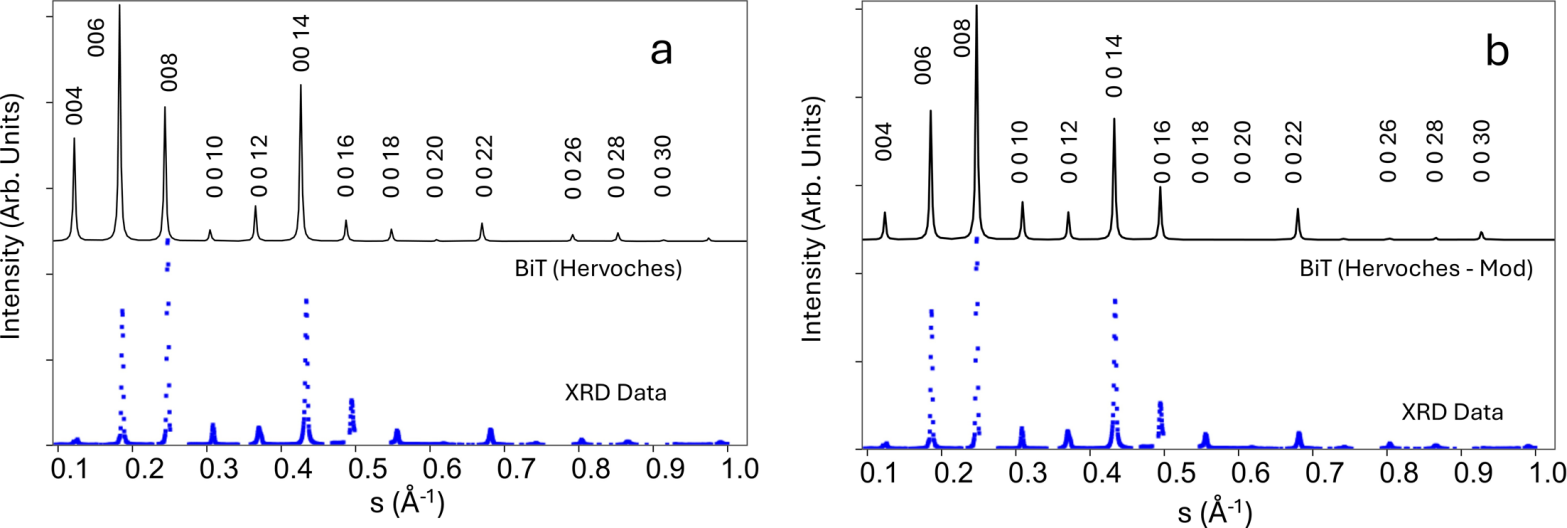
(*a*) Top (black): the diffraction profile for a 90%-oriented BiT film calculated using *CrystalMaker7* for the structural data reported by Hervoches & Lightfoot (1999 ▸) – the reflection peak Miller indices are indicated above the plot. Bottom (blue dots): the XRD data collected using monochromated Cu *K*α radiation from the epitaxial BiT thin film represented by the TEM images in Figs. 2 ▸ and S1. (The data have been edited to eliminate substrate peaks for clarity of presentation.) (*b*) Top (black): the diffraction profile for a 90%-oriented BiT film calculated using *CrystalMaker* with the modified Bi atomic positions presented in the main text. Bottom (blue dots): XRD data as for (*a*).

This 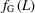 function was used in the OPB diffraction model represented by equations (5) and (6) to generate XRD intensity plots that were compared with experimental XRD data taken from the same sample as described above. The 25°C structural data published by Hervoches & Lightfoot (1999 ▸) were used as the initial basis for the intensity calculations. Fig. 9 ▸ presents the XRD data collected from the BiT sample when compared with various calculated profiles. Fig. 9 ▸(*a*) compares experimental XRD data with the diffraction profile for 90%-oriented BiT calculated using *CrystalMaker7* (https://crystalmaker.com/) with the structural data reported by Hervoches & Lightfoot (1999 ▸). The experimental XRD data points from the BiT film described in Fig. 2 ▸ are plotted as blue dots. (The substrate peaks have been removed for clarity.)

The profile calculated using the ‘Hervoches’ data (Hervoches & Lightfoot, 1999 ▸), which were taken from bulk material, compares quite poorly with the experimental data, both for the positions of the peaks and their relative intensities. In particular, the relative intensities of the 004, 006 and 008 peaks differ markedly from the experimental data, with the calculated 006 intensity being considerably greater than either 004 or 008, whereas for the experimental data, the 008 peak is the most intense. Accordingly, the *c*-axis lattice parameter and *c*-axis structural coordinates for the Bi atoms were adjusted manually to give the best fit with the observed intensity data for the whole diffraction profile, which was re-calculated using *CrystalMaker7*. (No attempt was made to adjust the Ti or O atomic positions as the structure factor is dominated by the contribution from the Bi atoms.) The revised profile (denoted ‘Hervoches-Mod’) is compared with the experimental XRD profile in Fig. 9 ▸(*b*), and there is now much better agreement for both peak positions and relative peak intensities. However, the diffraction profiles calculated using *CrystalMaker7* cannot reproduce the peak splitting observed in 004 and 0 0 12. The *c*-axis parameter was best fit at *c* = 32.35 Å, while the literature value is 32.8425 Å. The difference of about 1.5% is probably due to strain in the film. There was no sign of any systematic displacement errors in the peak positions. The literature values of the revised atomic coordinates (Hervoches & Lightfoot, 1999 ▸) are compared with the parameters derived from the model in Table 2 ▸. The coordinate shifts are ∼3% (Bi1) and 18% (Bi2) of the relevant Bi^3+^ ionic radius of 1.11 Å (Shannon & Prewitt, 1969 ▸). It is unsurprising that there are shifts in the average atomic positions relative to the ‘bulk’ structure, given the level of strain and the structural defectiveness of the film. The fact that by far the largest shift is in Bi2 is interesting, as this is the Bi atom that makes up the ‘fluorite’ layer separating the perovskite blocks, which one might expect to be more susceptible to shifts in a highly defective structure.

**Figure 10:**
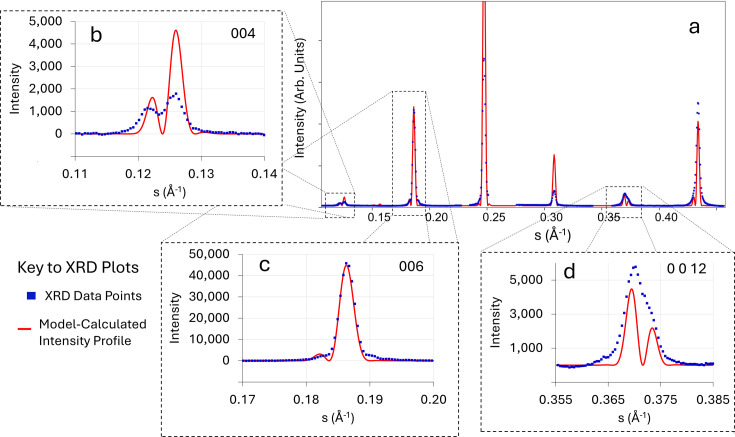
(*a*) The calculated diffracted intensity profile (in red) from the OPB model described in the main text, using the ‘modified Hervoches’ positions for the Bi atoms listed in Table 2 ▸, compared with the background-subtracted experimental XRD data points (blue dots) taken from the epitaxial BiT thin film represented by the TEM images in Figs. 2 ▸ and S1. Plots (*b*), (*c*) and (*d*) represent expanded views of the model/data comparison for the 004, 006 and 0 0 12 reflections, respectively.

The Hervoches-Mod structural data for the Bi atomic coordinates as listed in Table 2 ▸ and the revised *c*-axis lattice parameter can now be used in association with the new model presented here for the effects of OPBs on diffraction to assess the model’s applicability to the complete diffraction profile, including the split peaks. Fig. 10 ▸(*a*) shows (in red) a higher-resolution display of the diffraction profile calculated using the new model presented here [represented by equations (5) and (6)] compared with the experimental XRD data points from the BiT film described in Fig. 2 ▸ (again plotted as blue dots). (All the other structural data for the Ti and O atoms were kept constant, as for the Hervoches model). The distribution of inter-OPB lengths used for the model 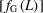 was as plotted in Fig. 8 ▸. The OPB model intensity data were generated using an average value of 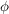
 = 37°, similar to that measured from the TEM images. The best-fit value of 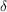 was 3.85 Å. This step height compares well to the STO *a*-axis parameter of 3.91 Å. This is significant as structural steps at the STO/film interface are thought to be the most likely source of OPBs in these films.

Figs. 10 ▸(*b*), 10 ▸(*c*) and 10 ▸(*d*) show expanded views of the XRD data points (in blue) compared with the model-calculated profile (in red). Figs. 10 ▸(*b*) and 10 ▸(*d*)] display the two main split peaks, 004 and 0 0 12, and Fig. 10 ▸(*c*) shows one non-split peak, 006, for comparison. The fit between the observations and the model is good for all the peaks. The model intensities have been scaled to give agreement with the experimental data for the 006 peak. The overall agreement between model profile and experimental data is much better than simply using the bulk Hervoches structural data, and the model successfully predicts the peak splitting. The predicted intensity is higher than the observations for the split 004 peak, and slightly lower for the 0 0 12 peak, but the predictions of peak splitting 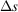 and the relative intensities of the two peaks within each split peak agree well for both the 004 and 0 0 12 reflections, which is very encouraging. Interestingly, the model predicts the shape of the 006 reflection very well, including the existence of a low-intensity shoulder on the low-*s* side of the peak, which is also seen in the experimental data. No attempt has been made to introduce other peak-broadening functions (to account for *e.g.* instrumental or strain broadening effects) into the model at this stage. The inclusion of a broadening term (*e.g.* Lorentzian) would probably allow the peak tails to be better fitted. However, that was not the main intention of this study.

## 4. Discussion

Overall, the predictions of the model represented by equations (5), (6) and (10) agree remarkably well with the observations, especially considering the simplicity of the structure presented in Fig. 4 ▸. No attempt has been made to use any refinement techniques (*e.g.* least-squares profile refinement methods) to optimize the crystal or micro/nano-structural parameters used in the model. In the case of the analysis of the SBTN peak splitting data presented in Fig. 7 ▸, the inputs to the model were the known structure from Shimakawa *et al.* (1999 ▸) together with the OPB displacement and OPB angle data from Zurbuchen *et al.* (2007 ▸). The model successfully predicts the dependence of XRD peak splitting on average OPB spacing and the results agree very well with the data reported in the Zurbuchen paper. In the case of the analysis of the effects on diffraction of OPBs in BiT thin films on STO represented by the data presented in Figs. 9 ▸ and 10 ▸, the inputs to the model were the observed distribution of inter-OPB lengths and the OPB angle 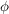, together with the initial guess for 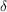 and the Hervoches-Mod structural coordinates and lattice parameters. The parameters 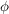 and 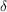 were adjusted manually to fit the model outputs with the data. There is good agreement between the observed and predicted overall XRD profiles, as well as the XRD peak splits and the relative intensities of the sub-peaks within the split Bragg maxima. This result gives confidence that the model, while structurally simple, provides a good analytical representation of the overall diffraction profile, the peak splitting 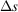 and the relative intensities of the sub-peaks within each split reflection in terms of physically relevant parameters that describe the characteristics of the OPBs. The model only considers the interference between the diffraction for the relatively displaced atomic planes on each side of the OPB. Hence, while it characterizes the nano­structure of the OPB well, it is not dependent upon the potential complexities of the structure in the immediate vicinity of the OPB, which represent a relatively small proportion of the diffracting volume when compared with the whole film (the region represented by the whole of the volume ABCD in Fig. 4 ▸). This is both an advantage, in that a relatively simple model can be used to say a good deal about the nanostructure of the samples, and a disadvantage, in that the complexities of the OPB itself can only be revealed by undertaking a full TEM examination of the sample.

A further advantage of the new model is that it has enabled, for the first time, a reliable prediction of which X-ray peaks will be doubled, given a knowledge of the displacement at the OPB (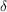). Perhaps more usefully, it is possible to invert this process so that a knowledge of which peaks are doubled can be used to derive an approximate value for 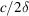. The resulting value of 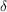 can then be used as an input parameter for a further adjustment of the model XRD profile parameters to fit the experimental data. It is considered that the application of a full least-squares profile refinement process to the data analysis in the context of the new model would assist with obtaining a still better fit between the observed and predicted diffraction patterns. However, the writing of the code for such a program goes well beyond the scope of this study, and in any case, such a program is not necessary to confirm the overall validity of the model, which has been amply demonstrated by the analysis presented here for the SBTN (*m* = 2) and BiT (*m* = 3) systems. While the model has only been applied here to structures with these two values of *m*, the theory is in no way limited by the value of *m* in the structure. It can be seen from Fig. 4 ▸ that the theory considers parallel blocks of material that are displaced relative to one another across the OPB. It is independent of the structural details within each block, the diffraction from which is described by the unit-cell structure factor 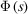 in equation (1). Hence, it will apply to any materials that exhibit OPBs of the type described (such as Ruddlesden–Popper phases), and its predictions will be independent of the value of *m* for the Aurivillius compounds. Preliminary analysis by the authors of BFTO films with *m* = 4 and *m* = 5, which also show split peaks, has indicated that the model can equally well be applied to these structures. However, the defect structures exhibited within these films are more complex than the SBTN and BiT films considered above. In addition to OPBs, there can be stacking faults (Halpin, 2021 ▸) and intergrowth regions with different values of *m* (Sun *et al.*, 2021 ▸). This all leads to considerable amounts of XRD peak broadening as well as splitting. A preliminary theoretical analysis of stacking fault defects along the lines of that presented here for OPBs has been performed, with promising results for the Aurivillius materials in systems for *m* > 3. However, as well as accounting for OPBs, a full theoretical description of the more complex structures that are obtained for the higher-*m* structures should also include the effects of stacking faults, inter-growths and arrays of these defects with statistical distributions of the parameters that define them, such as their physical size and variations in the OPB inclination angle 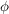 (Moore *et al.*, 2022 ▸). In the meantime, the theoretical model presented here for the OPBs in the simpler low-*m* structures is a useful contribution to the understanding of the way these defects affect diffraction and, conversely, it should facilitate the use of XRD to characterize the defects. It is also considered that the model is sufficiently general to be applicable to other forms of thin-film materials that exhibit OPBs, such as the Ruddlesden–Popper family.

## 5. Conclusions

A new analytical model has been developed that allows prediction of the OPB-engendered XRD peak doubling in epitaxial thin films of Aurivillius oxides. Other computer-based models (such as *DIFFaX*) have been used in the past to study the effects of stacking faults on layered materials. However, equation (5) is the first single fully analytical expression that accurately represents the XRD profile from Aurivillius films containing OPBs in terms of physically relevant parameters that describe the nanostructure of the OPBs. The average spacing of the OPBs and their statistical distribution, the structural displacement across the OPB, and the angle made by the OPB at the film–substrate interface can now be used in association with the unit-cell structure data to predict the diffraction profile. Given knowledge of these parameters, the model can be used to predict which XRD peaks will be split, the amount of splitting and the full XRD intensity profile. Conversely, it has been shown that, if reasonable guesses can be made for the relevant OPB parameters, an experimental XRD profile can be used to refine the parameter values to give a predicted profile that matches the experimental data much better than the profile predicted from the crystal structure alone, which does not predict the peak splitting. It has also been shown that the predictions of the model agree well with experimental observations for both the *m* = 2 Aurivillius material SBTN and the *m* = 3 material BiT. The model is applied here to Aurivillius oxides, but the derivation is general and it should also apply to other types of layered materials that show the presence of OPBs, such as the Ruddlesden–Popper family and high-temperature superconductors. The model is now presented to the layered-oxide community as a starting point to facilitate better understanding of nanostructures in layered materials.

## Supplementary Material

Supporting information. DOI: 10.1107/S1600576725004091/po5162sup1.pdf

## Figures and Tables

**Table 1:** Values of *l* as predicted from equation (8) for BiT and SBT when OPBs are present, for which a doubled peak is expected, compared with the values of *l* for which a doubled peak is observed

	BiT		SBT	
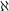	Predicted *l* for doubling from equation (8)	Observed *l* for doubling	Predicted *l* for doubling from equation (8)	Observed *l* for doubling
1	4.1	4	2.8	No 003 peak
3	12.3	12	8.3	8
5	20.5	20	13.9	14
7	28.7	No 0 0 29 peak	19.5	20

**Table 2:** Revised *Z* coordinates and occupancies for the Bi atoms used in the BiT (Hervoches-Mod) structure discussed in the main text

Atom	*Z* coordinate (revised)	*Z* coordinate (literature) (Hervoches & Lightfoot, 1999 ▸)	Atomic displacement (Å)
Bi1	0.0675	0.06639	−0.036
Bi2	0.205	0.21127	0.203

## References

[bb1] Aurivillius, B. (1949). *Ark. Kemi* **1**, 463–480.

[bb2] Aurivillius, B. (1950). *Ark. Kemi* **1**, 499–512.

[bb3] Bréger, J., Jiang, M., Dupré, N., Meng, Y. S., Shao-Horn, Y., Ceder, G. & Grey, C. P. (2005). *J. Solid State Chem.* **178**, 2575–2585.

[bb4] Campanini, M., Trassin, M., Ederer, C., Erni, R. & Rossell, M. D. (2019). *ACS Appl. Electron. Mater.* **1**, 1019–1028.

[bb5] Casas-Cabanas, M., Rodríguez-Carvajal, J. & Palacín, M. (2006). *Z. Kristallogr. Suppl.* **2006**, 243–248.

[bb6] Catalan, G., Seidel, J., Ramesh, R. & Scott, J. F. (2012). *Rev. Mod. Phys.* **84**, 119–156.

[bb7] Celeste, A., Tuccillo, M., Menon, A. S., Brant, W., Brandell, D., Pellegrini, V., Brescia, R., Silvestri, L. & Brutti, S. (2024). *Small Methods* **8**, 2301466. 10.1002/smtd.202301466 38164821

[bb8] Chu, Y. H., Cruz, M. P., Yang, C. H., Martin, L. W., Yang, P. L., Zhang, J. X., Lee, K., Yu, P., Chen, L. Q. & Ramesh, R. (2007). *Adv. Mater.* **19**, 2662–2666.

[bb9] Deepak, N., Zhang, P. F. F., Keeney, L., Pemble, M. E. & Whatmore, R. W. (2013). *J. Appl. Phys.* **113**, 187207.

[bb10] Dittrich, H. & Wohlfahrt-Mehrens, M. (2001). *Int. J. Inorg. Mater.* **3**, 1137–1142.

[bb11] Drits, V. A. & Tchoubar, C. (1990). *X-ray diffraction by disordered lamellar structures: theory and applications to microdivided silicates and carbons*, pp. 305–360. Springer Berlin Heidelberg.

[bb12] Faraz, A., Maity, T., Schmidt, M., Deepak, N., Roy, S., Pemble, M. E., Whatmore, R. W. & Keeney, L. (2017). *J. Am. Ceram. Soc.* **100**, 975–987.

[bb13] Fleck, E. E., Barone, M. R., Nair, H. P., Schreiber, N. J., Dawley, N. M., Schlom, D. G., Goodge, B. H. & Kourkoutis, L. F. (2022). *Nano Lett.* **22**, 10095–10101. 10.1021/acs.nanolett.2c03893 PMC9801418 36473700

[bb14] Garcia, V., Bibes, M., Bocher, L., Valencia, S., Kronast, F., Crassous, A., Moya, X., Enouz-Vedrenne, S., Gloter, A., Imhoff, D., Deranlot, C., Mathur, N. D., Fusil, S., Bouzehouane, K. & Barthélémy, A. (2010). *Science* **327**, 1106–1110. 10.1126/science.1184028 20075211

[bb15] Gradauskaite, E., Campanini, M., Biswas, B., Schneider, C. W., Fiebig, M., Rossell, M. D. & Trassin, M. (2020). *Adv. Mater. Interfaces* **7**, 2000202.

[bb16] Gradauskaite, E., Gray, N., Campanini, M., Rossell, M. D. & Trassin, M. (2021). *Chem. Mater.* **33**, 9439–9446.

[bb17] Gradauskaite, E., Hunnestad, K. A., Meier, Q. N., Meier, D. & Trassin, M. (2022). *Chem. Mater.* **34**, 6468–6475.

[bb18] Halpin, J. C. (2021). PhD thesis, University College Cork, Ireland.

[bb19] Hawley, M., Raistrick, I. D., Beery, J. G. & Houlton, R. J. (1991). *Science* **251**, 1587–1589. 10.1126/science.251.5001.1587 17793141

[bb20] Hervoches, C. H. & Lightfoot, P. (1999). *Chem. Mater.* **11**, 3359–3364.

[bb21] Holstein, W. L. (1993). *J. Appl. Phys.* **74**, 4963–4971.

[bb22] Keeney, L., Downing, C., Schmidt, M., Pemble, M. E., Nicolosi, V. & Whatmore, R. W. (2017). *Sci. Rep.* **7**, 1737. 10.1038/s41598-017-01902-1 PMC5431865 28496096

[bb23] Keeney, L., Maity, T., Schmidt, M., Amann, A., Deepak, N., Petkov, N., Roy, S., Pemble, M. E. & Whatmore, R. W. (2013). *J. Am. Ceram. Soc.* **96**, 2339–2357.

[bb24] Kelly, A. & Knowles, K. M. (2020). *Crystallography and crystal defects*, ch. 9. Wiley.

[bb25] Kim, J., Mun, J., Palomares García, C. M., Kim, B., Perry, R. S., Jo, Y., Im, H., Lee, H. G., Ko, E. K., Chang, S. H., Chung, S. B., Kim, M., Robinson, J. W. A., Yonezawa, S., Maeno, Y., Wang, L. & Noh, T. W. (2021). *Nano Lett.* **21**, 4185–4192. 10.1021/acs.nanolett.0c04963 33979525

[bb26] Kopp, V. S., Kaganer, V. M., Schwarzkopf, J., Waidick, F., Remmele, T., Kwasniewski, A. & Schmidbauer, M. (2012). *Acta Cryst.* A**68**, 148–155. 10.1107/S0108767311044874 22186291

[bb27] Lanson, B. (2011). *Layered mineral structures and their application in advanced technologies*, Vol. 11, pp. 151–202. European Mineralogical Union.

[bb28] Lee, H.-J., Soles, C. L. & Wu, W. (2011). *Opt. Express* **14**, 19573–19582.

[bb29] Leonardi, A. & Bish, D. L. (2020). *Inorg. Chem.* **59**, 5357–5367. 10.1021/acs.inorgchem.9b03464 32233425

[bb30] Li, L., Cheng, X., Jokisaari, J. R., Gao, P., Britson, J., Adamo, C., Heikes, C., Schlom, D. G., Chen, L.-Q. & Pan, X. (2018). *Phys. Rev. Lett.* **120**, 137602. 10.1103/PhysRevLett.120.137602 29694202

[bb31] MacLaren, I., Wang, L., Craven, A. J., Ramasse, Q. M., Schaffer, B., Kalantari, K. & Reaney, I. M. (2014). *APL Mater.* **2**, 066106.

[bb32] Mejri, C., Oueslati, W. & Amara, A. B. H. (2022). *E3S Web Conf.* **354**, 03009.

[bb33] Momma, K. & Izumi, F. (2011). *J. Appl. Cryst.* **44**, 1272–1276.

[bb34] Moore, K., O’Connell, E. N., Griffin, S. M., Downing, C., Colfer, L., Schmidt, M., Nicolosi, V., Bangert, U., Keeney, L. & Conroy, M. (2022). *Appl. Mater. Interfaces* **14**, 5525–5536. 10.1021/acsami.1c17383 PMC8815039 35044754

[bb35] Mundy, J. A., Brooks, C. M., Holtz, M. E., Moyer, J. A., Das, H., Rébola, A. F., Heron, J. T., Clarkson, J. D., Disseler, S. M., Liu, Z., Farhan, A., Held, R., Hovden, R., Padgett, E., Mao, Q., Paik, H., Misra, R., Kourkoutis, L. F., Arenholz, E., Scholl, A., Borchers, J. A., Ratcliff, W. D., Ramesh, R., Fennie, C. J., Schiffer, P., Muller, D. A. & Schlom, D. G. (2016). *Nature* **537**, 523–527. 10.1038/nature19343 27652564

[bb36] Nakajima, H., Kurushima, K., Mine, S., Tsukasaki, H., Matsuoka, M., Gao, B., Cheong, S.-W. & Mori, S. (2021). *Commun. Mater.* **2**, 109.

[bb37] Narayan, S., Joshi, V., McMillan, L. & De Araujo, C. P. (1999). *Integr. Ferroelectr.* **25**, 169–177.

[bb38] O’Mahony, S. M., Ren, W., Chen, W., Chong, Y. X., Liu, X., Eisaki, H., Uchida, S., Hamidian, M. H. & Davis, J. C. S. (2022). *Proc. Natl Acad. Sci. USA* **119**, e2207449119. 10.1073/pnas.2207449119 PMC9477408 36067325

[bb39] Oueslati, W., Mejri, C. & Amara, A. B. H. (2022). *Nanoclay – recent advances, new perspectives and applications*. IntechOpen.

[bb40] Pitcher, M. J., Mandal, P., Dyer, M. S., Alaria, J., Borisov, P., Niu, H., Claridge, J. B. & Rosseinsky, M. J. (2015). *Science* **347**, 420–424. 10.1126/science.1262118 25613888

[bb41] Pomiro, F., Ablitt, C., Bristowe, N. C., Mostofi, A. A., Won, C., Cheong, S.-W. & Senn, M. S. (2020). *Phys. Rev. B* **102**, 014101.

[bb42] Ramesh, T. N., Jayashree, R. S. & Kamath, P. V. (2003). *Clays Clay Miner.* **51**, 570–576.

[bb43] Schmidt, M., Amann, A., Keeney, L., Pemble, M. E., Holmes, J. D., Petkov, N. & Whatmore, R. W. (2014). *Sci. Rep.* **4**, 5712. 10.1038/srep05712 PMC4100018 25026969

[bb44] Scott, J. F. & Paz de Araujo, C. A. (1989). *Science* **246**, 1400–1405. 10.1126/science.246.4936.1400 17755995

[bb45] Shannon, R. D. & Prewitt, C. T. (1969). *Acta Cryst.* B**25**, 925–946.

[bb46] Shimakawa, Y., Kubo, Y., Nakagawa, Y., Kamiyama, T., Asano, H. & Izumi, F. (1999). *Appl. Phys. Lett.* **74**, 1904–1906.

[bb47] Subbarao, E. C. (1961). *Phys. Rev.* **122**, 804–807.

[bb48] Sun, S., Li, Y., Yin, X., Liu, C., Li, X., Ti, R., Fang, L., Zhang, T., Peng, R. & Lu, Y. (2021). *J. Mater. Chem. C.* **9**, 4825–4837.

[bb49] Taylor, G. W. & Miller, A. (1970). *Proc. IEEE* **58**, 1220–1229.

[bb50] Tian, G., Yang, W., Chen, D., Fan, Z., Hou, Z., Alexe, M. & Gao, X. (2019). *Natl Sci. Rev.* **6**, 684–702. 10.1093/nsr/nwz100 PMC8291546 34691923

[bb51] Treacy, M., Newsam, J. & Deem, M. (1991). *Proc. R. Soc. London Ser. A* **433**, 499–520.

[bb52] Van Uitert, L. G. & Egerton, L. (1961). *J. Appl. Phys.* **32**, 959.

[bb53] Wang, Q., Wang, C.-M., Wang, J.-F. & Zhang, S. (2016). *Ceram. Int.* **42**, 6993–7000.

[bb54] Wang, Q., Zhang, X., Wang, C. J., Zhu, J., Guo, Z. & O’Hare, D. (2012). *J. Mater. Chem.* **22**, 19113–19121.

[bb55] Weber, M. C., Zemp, Y., Trassin, M., Simonov, A., Schaab, J., Gao, B., Cheong, S.-W., Lottermoser, T. & Fiebig, M. (2022). *Adv. Elect Mater.* **8**, 2100434.

[bb56] Yang, F., Tang, M. H., Ye, Z., Zhou, Y. C., Zheng, X. J., Tang, J. X., Zhang, J. J. & He, J. (2007). *J. Appl. Phys.* **102**, 044504.

[bb57] Yuan, H. & Bish, D. L. (2019). *Clays Clay Miner.* **67**, 399–409.

[bb58] Zheng, H., Wang, J., Xu, Z. & Gui, J. (2014). *J. Appl. Cryst.* **47**, 879–886.

[bb59] Zurbuchen, M. A., Tian, W., Pan, X. Q., Fong, D., Streiffer, S. K., Hawley, M. E., Lettieri, J., Jia, Y., Asayama, G., Fulk, S. J., Comstock, D. J., Knapp, S., Carim, A. H. & Schlom, D. G. (2007). *J. Mater. Res.* **22**, 1439–1471.

