# Supplementary material for: A model for out-of-phase boundary induced X-ray diffraction peak profile changes in Aurivillius oxide thin films
Source: J Appl Crystallogr. 2025 Jul 2;58(Pt 4):1191–204. doi: 10.1107/S1600576725004091 (PMC12321024; doi:10.1107/S1600576725004091)
Supplement: Supplementary file 1 [file j-58-01191-sup1.pdf]

JOURNAL OF APPLIED CRYSTALLOGRAPHY

Volume 58 (2025)

Supporting information for article:

A model for out-of-phase boundary induced X-ray diffraction peak profile changes in Aurivillius oxide thin films

Roger W. Whatmore, Debismita Dutta and Lynette Keeney

S1. Simplifying the Expression for $\Gamma_N(s)$

In the Main Text, we have equation (4) as:

$$\Gamma_N(s) = \frac{2}{L}\left\{\sum_{p=-n}^{p=+n} \zeta_p cos\left(2\pi h_p s\right)\right\} \quad \text{(S1)}$$

We now derive expressions for ζ_p and h_p by referring to Table S1, which tabulates h_p for each layer p on the undisplaced side (ABC) of the block.

Table S1 Tabulation of the values of h_p for selected layers p in region ABC of Fig. 4 (b) in the (Main Text).

Layer (p)	-5	-4	-1
h_p	$-\{(4+½)c+\varepsilon\}$	$-\{(3½)c+\varepsilon\}$	$-\{c/2+\varepsilon\}$
Layer (p)	0	1	5
h_p	$\{c/2-\varepsilon\}$	$\{(1½)c+\varepsilon\}$	$\{(5½)c+\varepsilon\}$

We can write a general expression for h_p as:

$$h_p = \{(p+½)c-\varepsilon\} \quad \text{(S2)}$$

To derive an expression for ζ_p, we can write:

$\zeta_p = \frac{L}{2} - h_p cot\phi$ (This recognises the fact that for $p < 0$, $h_{-p} < 0$.)

Substituting for h_p from equation (S2), we have:

$$\zeta_p = \frac{L}{2} - \{(p+½)c-\varepsilon\}cot\phi \quad \text{(S3)}$$

So, we can now substitute for h_p and ζ_p from equations (S2) and (S3) into equation (4) to obtain:

$$\Gamma_N(s) = \frac{2}{L}\left\{\sum_{p=-n}^{p=+n}\left(\frac{L}{2} - \{(p+½)c-\varepsilon\}cot\phi\right) cos(2\pi\{(p+½)c-\varepsilon\}s)\right\}$$

We have: $L = \Delta^{-1}$, so:

$$\Gamma_N(s) = \sum_{p=-n}^{p=+n}(1-2\Delta\{(p+½)c-\varepsilon\}cot\phi)cos(2\pi\{(p+½)c-\varepsilon\}s) \quad \text{(S4)}$$

If we look at the terms in the summation in equation (S4), we can write:

$$\Gamma_N(s) = \sum_{p=-n}^{p=+n} X_p \quad \text{where } N = (2n+1) \quad \text{(S5)}$$

$$X_p = (1-2\Delta\{(p+½)c-\varepsilon\}cot\phi)cos(2\pi\{(p+½)c-\varepsilon\}s) \quad \text{(S6)}$$

Put

$Z = \Delta cot\phi$

Then

$$X_p = (1-2Z\{(p+½)c-\varepsilon\})cos(2\pi\{(p+½)c-\varepsilon\}s)$$

If we consider an example where: $n = 3$ and $N = (2n+1) = 7$, and recognising that $\varepsilon = \frac{\delta}{2}$ then we can tabulate the terms for X_p as follows (Table S2).

Table S2 Terms from equation (S6).

p	X_p	Expression (1) →	→ Expression (2)
-3	X_{-3}	$\left(1-2\mathrm{Z}\left\{\frac{-5c}{2}-\frac{\delta}{2}\right\}\right)cos\left(2\pi\left\{\frac{-5c}{2}-\frac{\delta}{2}\right\}s\right)$	$(1+\mathrm{Z}\{5c+\delta\})cos(\pi\{5c+\delta\}s)$
-2	X_{-2}	$\left(1-2\mathrm{Z}\left\{\frac{-3c}{2}-\frac{\delta}{2}\right\}\right)cos\left(2\pi\left\{\frac{-3c}{2}-\frac{\delta}{2}\right\}s\right)$	$(1+\mathrm{Z}\{3c+\delta\})cos(\pi\{3c+\delta\}s)$
-1	X_{-1}	$\left(1-2\mathrm{Z}\left\{\frac{-c}{2}-\frac{\delta}{2}\right\}\right)cos\left(2\pi\left\{\frac{-c}{2}-\frac{\delta}{2}\right\}s\right)$	$(1+\mathrm{Z}\{c+\delta\})cos(\pi\{c+\delta\}s)$
0	X_0	$\left(1-2\mathrm{Z}\left\{\frac{c}{2}-\frac{\delta}{2}\right\}\right)cos\left(2\pi\left\{\frac{c}{2}-\frac{\delta}{2}\right\}s\right)$	$(1-\mathrm{Z}\{c-\delta\})cos(\pi\{c-\delta\}s)$
1	X_1	$\left(1-2\mathrm{Z}\left\{\frac{3c}{2}-\frac{\delta}{2}\right\}\right)cos\left(2\pi\left\{\frac{3c}{2}-\frac{\delta}{2}\right\}s\right)$	$(1-\mathrm{Z}\{3c-\delta\})cos(\pi\{3c-\delta\}s)$
2	X_2	$\left(1-2\mathrm{Z}\left\{\frac{5c}{2}-\frac{\delta}{2}\right\}\right)cos\left(2\pi\left\{\frac{5c}{2}-\frac{\delta}{2}\right\}s\right)$	$(1-\mathrm{Z}\{5c-\delta\})cos(\pi\{5c-\delta\}s)$
3	X_3	$\left(1-2\mathrm{Z}\left\{\frac{7c}{2}-\frac{\delta}{2}\right\}\right)cos\left(2\pi\left\{\frac{7c}{2}-\frac{\delta}{2}\right\}s\right)$	$(1-\mathrm{Z}\{7c-\delta\})cos(\pi\{7c-\delta\}s)$

It is easy to see that the terms in the summation in equation (S6) represented in Table S2 under the column labelled Expression (2) can be grouped together by the p values: p =-1 with p =0, p =-2 with p =1 , and p =-3 with p =2, with p =3 on its own. Using the trigonometric identities:

$$cos(A+B)+cos(A-B)=2cosA\,.\,cosB$$

and

$$cos(A+B)-cos(A-B)=-2sinA\,.\,sinB$$

it can be shown that:

$$\Gamma_7(s)=2.\left[(1+\zeta\delta)cos(\pi\delta s)\{cos(\pi cs)+cos(3\pi cs)+cos(5\pi cs)\}-\zeta csin(\pi\delta s)\{sin(\pi cs)+3sin(3\pi cs)+5sin(5\pi cs)\}\right]+(1-\zeta\{7c-\delta\})cos(\pi\{7c-\delta\}s) \quad \text{(S7)}$$

We can generalise this expression to any value of N, as:

$$\Gamma_N(s)=2\begin{bmatrix}(1+\mathrm{Z}\delta)cos(\pi\delta s)\sum_{k=1}^{n}cos\left((2k-1)\pi cs\right)-\\ \mathrm{Z}c.\,sin(\pi\delta s)\sum_{k=1}^{n}(2k-1).\,sin((2k-1)\pi cs)\end{bmatrix}+(1-\mathrm{Z}\{Nc-\delta\})cos(\pi\{Nc-\delta\}s) \quad \text{(S8)}$$

This can be further simplified, as it can be shown that:

$$\sum_{k=1}^{n}cos((2k-1)\pi cs)=\frac{\sin(n\pi cs)}{sin(\pi cs)}\cos(n\pi cs)=\frac{\sin(2n\pi cs)}{2sin(\pi cs)}$$

So, equation (S8) becomes:

$$\Gamma_N(s) = 2\begin{bmatrix} (1+\mathrm{Z}\delta)cos(\pi\delta s)\dfrac{\sin(2n\pi cs)}{2sin(\pi cs)} \\ -\mathrm{Z}c.\,sin(\pi\delta s)\displaystyle\sum_{k=1}^{n}(2k-1).\,sin\big((2k-1)\pi cs\big) \end{bmatrix} + (1-\mathrm{Z}\{Nc-\delta\})cos(\pi\{Nc-\delta\}s) \quad \text{(S9)}$$

It can be shown that:

$$\sum_{k=1}^{n}(2k-1).\,sin\big((2k-1)\pi cs\big)$$

$$= cosecant(\pi cs)\left\{n[sin^2(n\pi cs) - cos^2(n\pi cs)] + \frac{1}{2}cot(\pi cs)sin(2n\pi cs)\right\}$$

$$= \frac{1}{sin(\pi cs)}\left\{-ncos(2n\pi cs) + \frac{cot(\pi cs)}{2}sin(2n\pi cs)\right\} = \left\{\frac{cot(\pi cs)sin(2n\pi cs)}{2sin(\pi cs)} - \frac{ncos(2n\pi cs)}{sin(\pi cs)}\right\}$$

So:

$$\sum_{k=1}^{n}(2k-1).\,sin\big((2k-1)\pi cs\big) = \left\{\frac{cot(\pi cs)sin(2n\pi cs)}{2sin(\pi cs)} - \frac{ncos(2n\pi cs)}{sin(\pi cs)}\right\}$$

And as we have $Nc = Ltan\phi$, equation (S9) can be rewritten as:

$$\Gamma_N(s) = 2\begin{bmatrix} (1+\mathrm{Z}\delta)cos(\pi\delta s)\frac{\sin(2n\pi cs)}{2sin(\pi cs)} \\ -\mathrm{Z}c.\,sin(\pi\delta s)\left\{\frac{cot(\pi cs)sin(2n\pi cs)}{2sin(\pi cs)} - \frac{ncos(2n\pi cs)}{sin(\pi cs)}\right\} \end{bmatrix} + (1-\mathrm{Z}\{Ltan\phi-\delta\})cos(\pi\{Ltan\phi-\delta\}s) \quad \text{(S10)}$$

S2. TEM Images of BiT Thin Films

Fig. S1 presents three further dark-field, cross-sectional TEM images taken from the same BiT thin film sample as was used to derive the TEM and XRD data presented in Figs. 2 and 8 of the main text.

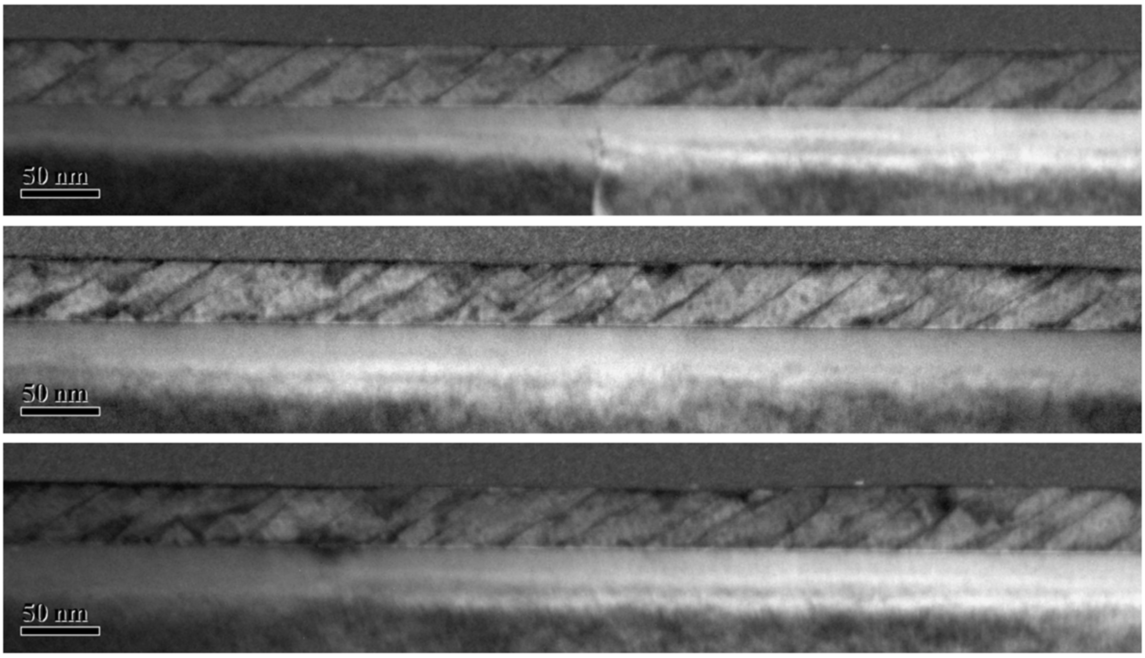

Figure S1 Dark-field cross sectional TEM images taken from the BiT sample used to generate the TEM images and RXD data presented in Figs. 2 and 8 of the main text.
